# Supplementary material for: The Evolution and Characterization of the RNA Interference Pathways in Lophotrochozoa
Source: Genome Biol Evol. 2024 May 7;16(5):evae098. doi: 10.1093/gbe/evae098 (PMC11114477; doi:10.1093/gbe/evae098)
Supplement: evae098_Supplementary_Data [file evae098_supplementary_data.zip › Supplementary_materials.zip!/selected_miRNAs/NoGe_selected_miRNAs/known_miRNA_families_pdf_files/NMRB01000754.1_Notospermus_geniculatus_strain_Ushimado_scaffold754,_whole_genome_shotgun_sequence_20344.pdf]

Provisional ID	:	NMRB01000754.1_Notospermus_geniculatus_strain_Ushimado_scaffold754,_whole_genome_shotgun_sequence_20344
Score total	:	46794.8
Score for star read(s)	:	3.9
Score for read counts	:	46786.3
Score for mfe	:	1.7
Score for randfold	:	0
Score for cons. seed	:	3
Total read count	:	91781
Mature read count	:	91084
Loop read count	:	0
Star read count	:	697

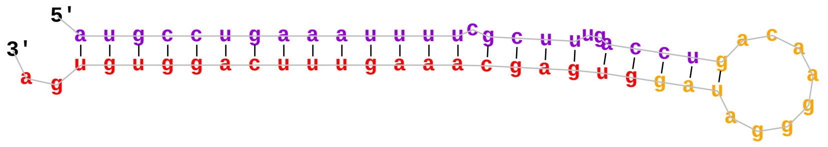

Genome coordinates: NMRB01000754.1 77223-77282

pre-miRNA sequence:
AUGCCUGAAAUUUUCGCUUUGACCUAUGCCUGAAAUUUUCGCUUUGACCUGACAAGGGAUAGGUGAGCAAAGUUUCAGGUGUGA

Mature sequence: GUGAGCAAAGUUUCAGGUGUGA

Aligned mature sequence of the *N. geniculatus* pre-miRNA with the top 5 hits from the BLAST search against the pre-miRNA mirGeneDB set:

```
                            ********************
Nge_Mir-87-P1               gugagcaaaguuucagguguga
Isc-Mir-87-P2_3p            gugagcaaaguuucaggugugu
Lan-Mir-87-P2_3p            gugagcaaaguuucaggugugu
Lpo-Mir-87-P2h_3p           gugagcaaaguuucaggugugu
Csc-Mir-87-P2l_3p           gugagcaaaguuucaggugugu
Lgi-Mir-87-P1_3p            gugagcaaaguuucagguguau
```
